# Identifying occult bladder outlet obstruction in women with detrusor-underactivity-like urodynamic profiles

**DOI:** 10.1038/s41598-021-02617-0

**Published:** 2021-12-01

**Authors:** Po-Ming Chow, Sheng-Mou Hsiao, Hann-Chorng Kuo

**Affiliations:** 1 Department of Urology, National Taiwan University Hospital and College of Medicine, No. 7, Chung-Shan South Road, Taipei, 100 Taiwan; 2 Department of Obstetrics and Gynecology, Far Eastern Memorial Hospital, Banqiao, No.21, Sec. 2, Nanya S. Rd., Banqiao Dist., New Taipei City, 220 Taiwan; 3 Department of Obstetrics and Gynecology, National Taiwan University Hospital and College of Medicine, Taipei, Taiwan; 4 Graduate School of Biotechnology and Bioengineering, Yuan Ze University, Taoyuan, Taiwan; 5 Department of Urology, Hualien Tzu Chi Hospital, Buddhist Tzu Chi Medical Foundation and Tzu Chi University, 707, Sec. 3, Chung-Yang Rd., Hualien, Taiwan

**Keywords:** Urology, Bladder

## Abstract

Voiding dysfunction can result from detrusor underactivity (DU), bladder outlet obstruction (BOO), or both. Conceptually, women with high-pressure low-flow urodynamic profiles are diagnosed with BOO without DU. However, the possibility of BOO is often neglected in women with DU-like (low-pressure low-flow) urodynamic (UDS) profiles. By reviewing the videourodynamic studies (VUDS) of 1678 women, our study identified the key factors suggesting urodynamic BOO (determined by radiographic evidence of obstruction) in women with DU-like UDS profiles (Pdet.Qmax < 20 cmH2O and Qmax < 15 mL/s). In 355 women with DU-like UDS profiles, there were 70 (19.7%) with BOO and 285 (80.3%) without BOO. The BOO group had predominantly obstructive symptoms. The BOO group showed significantly decreased bladder sensation, lower detrusor pressure (Pdet.Qmax), lower flow rate (Qmax), smaller voided volume, and larger post-voiding residual (PVR) compared to the non-BOO group. In multivariate analysis, volume at first sensation, Qmax, PVR, and detrusor overactivity (DO) remained independent factors for BOO. The receiver operating characteristic (ROC) areas for the parameters were largest for PVR (area = 0.786) and Qmax (area = 0.742). The best cut-off points were 220 mL for PVR and 4 mL/s for Qmax. Our findings provide simple indicators for BOO in women with DU.

## Introduction

The lower urinary tract has two functions: storage and voiding. Either storage or voiding dysfunction may result in lower urinary tract symptoms (LUTS). In both men and women, storage symptoms include frequency, urgency, nocturia, and incontinence; and voiding symptoms include weak stream, intermittency, hesitancy, straining, and sensation of incomplete voiding. Although the symptoms are classified as storage or voiding, the underlying etiologies are often intertwined, and the pathophysiology is suggestive at best.

Normal voiding requires good contractility from the detrusor muscle and low resistance from the bladder outlet. Therefore, voiding dysfunction can result from detrusor underactivity (DU), bladder outlet obstruction (BOO), or both^1^. DU has been defined by the International Continent Society (ICS) in 2002 as “a contraction of reduced strength and/or duration, resulting in prolonged bladder emptying and/or failure to achieve complete bladder emptying within a normal time span^2^.” Although the definition considers the bladder aspect of voiding dysfunction, no specific parameter is included.

To address the association between overactive bladder (OAB) and detrusor overactivity (DO), the most recent term “underactive bladder” (UAB) has been defined in a consensus on behalf of the ICS. The consensus states that “UAB is characterized by a slow urinary stream, hesitancy, and straining to void, with or without a feeling of incomplete bladder emptying sometimes with storage symptoms^3^.” This definition of UAB comprises the essential symptoms of BOO, making the two terms indistinguishable from each other. Although the symptoms might be overlapping between DU and BOO, the treatment strategies are different.

Bladder contractility index (BCI) and bladder outlet obstruction index (BOOI) are widely accepted terms in diagnosing male voiding dysfunction^4^. Unfortunately, there are no standard urodynamic criteria for the diagnosis of DU and BOO in women. Conceptually, women with high-pressure low-flow urodynamic profiles are diagnosed with BOO without DU. However, the possibility of BOO is often neglected in women with DU-like (low-pressure low-flow) urodynamic profiles. Identification of urodynamic BOO provides these patients with additional treatment options, such as transurethral incision of the bladder neck (TUI-BN) or urethral botulinum toxin injection, and a better chance of spontaneous urination. VUDS is a good method to detect BOO in such patients^5^. Due to its invasive nature and the exposure to radiation, VUDS is not routinely performed in all women with voiding dysfunction, thereby resulting in misdiagnosis and under-treatment. By reviewing a large database of VUDS, we intended to identify the key factors suggesting urodynamic BOO in women with DU-like UDS profile.

## Materials and methods

### Ethical approval

The Institutional Review Board and Ethics Committee of Hualien Tzu Chi Hospital approved this study. The IRB number was 100–06. The study followed the principles of the Declaration of Helsinki. Patients’ informed consents were waived by the Institutional Review Board and Ethics Committee of the Hualien Tzu Chi Hospital due to the retrospective nature of this study.

### Inclusion and exclusion criteria

Between November 1997 and September 2019, all women with lower urinary tract symptoms visiting the medical center for treatment were reviewed. Patients who had DU-like UDS profiles (Pdet.Qmax < 20 cmH2O and Qmax < 15 mL/s)^6,7^ were included for analysis. Patients who had spinal cord injury, multiple sclerosis, myelomeningocele, stress urinary incontinence, potential mechanical obstruction, interstitial cystitis, or ketamine cystitis were excluded.

### VUDS definitions

The VUDS parameters were recorded at the time of the examination. Bladder sensation was represented by volumes at first sensation of bladder filling (FSBF), normal desire (ND), and urgency (U). Bladder capacity was calculated by adding post-voiding residual (PVR) to voided volume. Bladder compliance was calculated using pressure and volume at normal desire (ND). Voiding efficiency (VE) was calculated by dividing voided volume by bladder capacity. Maximal flow rate (Qmax) and detrusor pressure at maximum flow rate (Pdet.Qmax) were detected during pressure-flow study. Bladder outlet obstruction index (BOOI) was calculated using (Pdet.Qmax – 2 × Qmax). Detrusor overactivity (DO) was defined as any detrusor contraction which has been symptomatic by the patient during the filling phase of cystometry. BOO was determined by radiographic evidence of obstruction between the bladder neck and distal urethra in the presence of a sustained detrusor contraction^5^. Patients who do not have open bladder necks during abdominal straining while attempting to void were classified as having urodynamic BOO.

### Outcome assessment

The primary outcome was the differences of UDS parameters between the BOO and the non-BOO groups. The secondary outcomes were the factors suggestive of BOO. R version 4.0.0 was used for statistical analyses, which included Student’s t-test for numerical data and Chi-square test for categorical data. A generalized linear model was used to generate the odds ratio and p-value for the predicting factors for BOO. Receiver operating characteristic (ROC) analyses were used to evaluate the diagnostic accuracy of each factor, and Youden’s index was used to determine the best cut-off value. All statistical tests were two-tailed, with p < 0.05 indicating significance.

## Results

**Figure 1:**
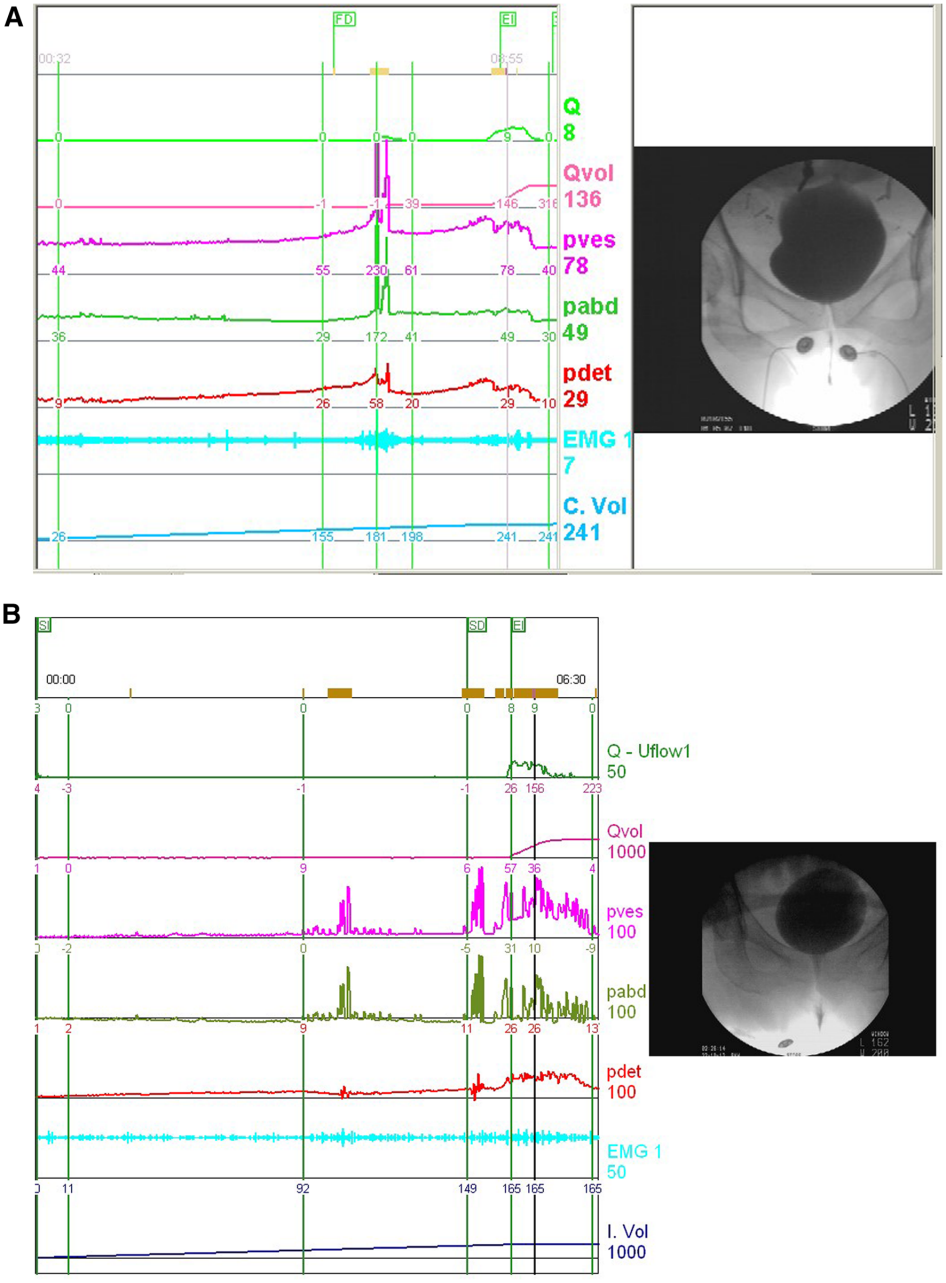
(**A**) Detrusor underactivity in a woman with bladder neck obstruction. The bladder neck was not widely open during the urination attempt. After receiving transurethral incision of the bladder neck, she resumed spontaneous voiding. (**B**) Detrusor underactivity in a woman without bladder outlet obstruction. The patient used abdominal pressure to aid spontaneous urination.

After reviewing 1678 cases of VUDS, 355 women with DU-like UDS profile were included in this study. The mean age was 68.5 ± 14.9 year old. There were 70 (19.7%) with BOO and 285 (80.3%) without BOO. The mean age was 68.6 ± 15.0 and 68.5 ± 14.9, respectively. In the BOO group, there were 2 patients with stroke and 1 patient with diabetes. In the non-BOO group, there were 19 patients with stroke, 8 patients with diabetes, 4 patients with dementia, and 1 patient with end-stage renal disease. Representative VUDS findings of DU patients with BOO and non-BOO are featured in Fig. 1.

The prevalence of symptoms was listed in Table 1. The BOO group had significantly more obstructive symptoms, including difficult voiding (79% vs. 38%) and urinary retention (11% vs. 4%), compared to the non-BOO group. In addition, the BOO group had significantly fewer storage symptoms, including frequency (40% vs. 69%) and urgency (36% vs. 64%).

**Table 1:** Symptoms of women with or without BOO.

Obstruction	Yes (n = 70)		No (n = 285)		p-value
	N	%	N	%	
Frequency	28	40%	198	69%	0.0000*
Urgency	25	36%	182	64%	0.0000*
Nocturia	0	0%	4	1%	0.3189
Difficult voiding	55	79%	107	38%	0.0000*
AUR	8	11%	10	4%	0.0068*
Recurrent UTI	6	9%	15	5%	0.2931

*p-value < 0.05

In the 70 cases with obstruction, 42 (60%) were identified to have obstruction levels at bladder neck, and 28 (40%) below the bladder neck. The detailed UDS profiles were listed in Table 2. During the filling phase, the BOO group had significantly larger bladder volumes at ND than the non-BOO group (253 mL vs. 215 mL), as well as larger volume at U (318 mL vs. 240 mL). Bladder capacity was significantly larger (418 mL vs. 298 mL). The prevalence of DO was significantly lower in the BOO group (10% vs. 42.8%). Bladder compliance was similar between the two groups. During the voiding phase, the BOO group had significantly lower Pdet.Qmax (6.6 cmH2O vs. 10.1 cmH2O) and Qmax (3.5 mL/s vs. 7.4 mL/s), but higher BOOI (-0.41 vs. -4.64). The BOO group had significantly smaller voided volume (87.9 mL vs. 168.1 mL), larger PVR (335.3 mL vs. 134.7 mL), and therefore smaller VE (0.25 vs. 0.59).

**Table 2:** UDS parameters of women with or without BOO.

Obstruction	Yes (n = 70)		No (n = 285)		p-value
	Mean	SD	Mean	SD	
FSBF	153.1	60.6	138.5	80.5	0.1027
ND	253.9	101.9	215.5	118.8	0.0088*
U	318.8	115.3	240.6	125.9	0.0000*
Compliance	63.6	54.8	75.1	87.3	0.1720
Pdet.Qmax	6.6	6.4	10.1	5.7	0.0001*
Qmax	3.5	4.5	7.4	4.3	0.0000*
Volume	87.9	117.6	168.1	127.5	0.0000*
PVR	335.3	221.0	134.7	156.3	0.0000*
Capacity	418.5	193.9	298.4	158.1	0.0000*
VE	0.25	0.33	0.59	0.36	0.0000*
BOOI	− 0.41	8.26	− 4.64	9	0.0004*
DO	7 (10%)		122 (42.8%)		0.0000*

*p-value < 0.05

**Figure 2:**
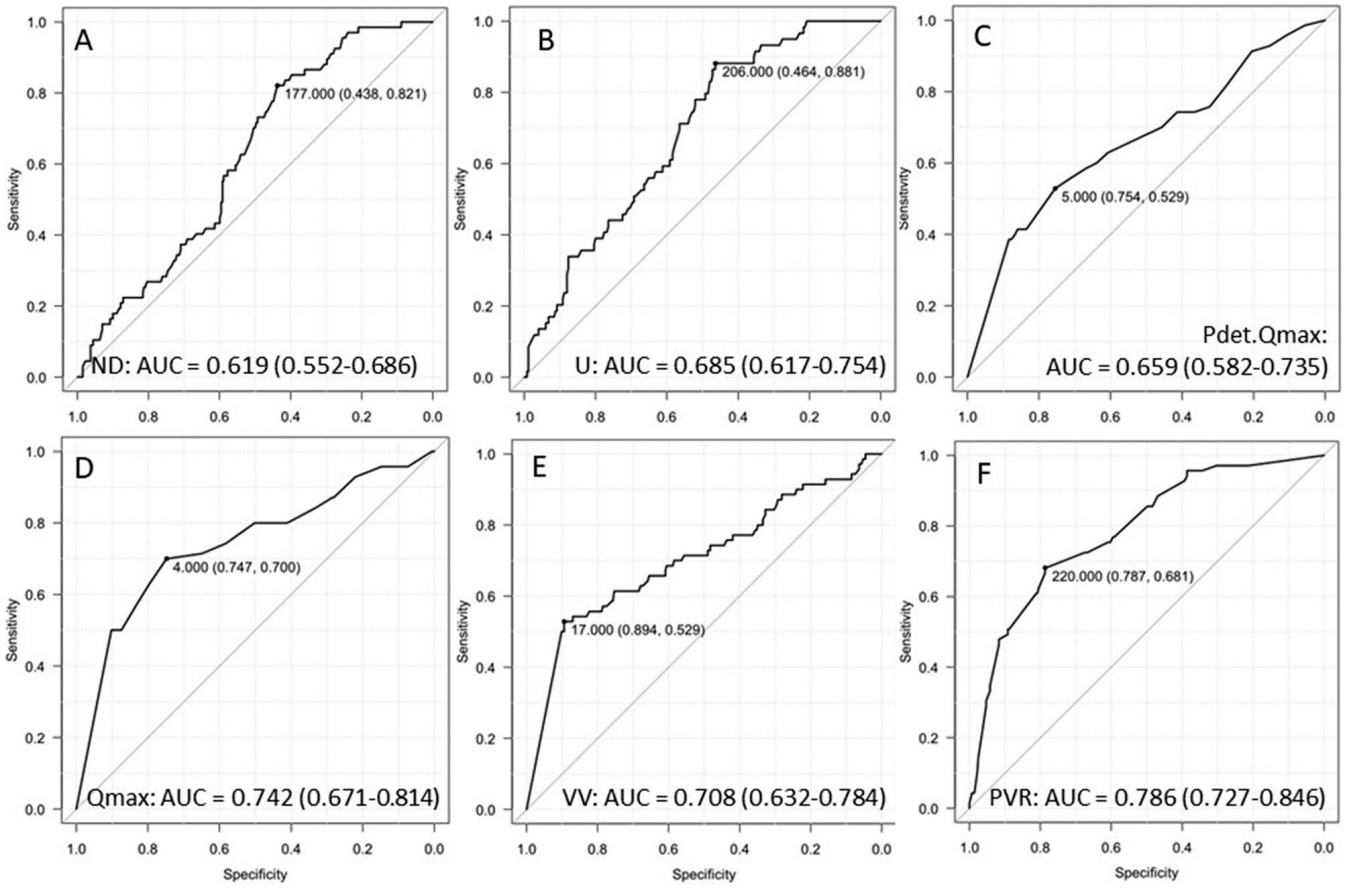
ROC curves for predicting factors for BOO: **(A)** normal desire, **(B)** urgency, **(C)** Pdet.Qmax, **(D)** Qmax, **(E)** voided volume, **(F)** PVR.

The significant UDS parameters were included in a regression model (Table 3). In multivariate analysis, ND, Qmax, PVR, and DO remained independent factors for BOO. The numerical parameters were analyzed with ROC curves (Fig. 2). The areas under the curve (AUC) for the parameters are largest for PVR (AUC = 0.786) and Qmax (AUC = 0.742). The best cut-off values were 220 mL for PVR and 4 mL/s for Qmax.

**Table 3:** Multivariate analysis for UDS parameters related to BOO.

	OR	Lower 95%CI	Upper 95%CI	p-value
ND	0.99	0.98	1.00	0.0098*
U	1.01	1.01	1.01	0.1380
Pdet.Qmax	1.01	0.95	1.08	0.7940
Qmax	0.86	0.75	0.99	0.0404*
VV	1.00	1.00	1.01	0.4330
PVR	1.01	1.00	1.01	0.0000*
DO	0.24	0.09	0.66	0.0055*

*p-value < 0.05

## Discussion

In this large cohort of women with DU-like UDS profiles, the prevalence of BOO was 19.7%. The BOO group had distinct symptoms and UDS findings from the non-BOO group. There were more AUR in the BOO group and more frequency and urgency in the non-BOO group. As for the UDS profiles, there were poorer bladder sensation, lower Pdet.Qmax, lower Qmax, smaller VV, larger PVR, and fewer DO in the BOO group. These UDS findings suggest profound detrusor damage or areflexia and impending bladder failures in the BOO group.

The true prevalence of BOO in women varies widely from 2.7 to 23% among different studies^8^. In a recent prospective VUDS study consisting of 1142 women with LUTS, 19% were diagnosed with BOO^9^. However, a large proportion of cases in this study had prior anti-incontinence surgery, suggesting iatrogenic BOO. In another prospective UDS study of 792 women with LUTS, the prevalence of voiding dysfunction was 12.8%, of which 87.2% were diagnosed with BOO^10^. In our cohort, the prevalence of BOO was relatively high (19%) compared to other reports. As persistent, untreated BOO is an important cause of DU^11^, it is reasonable that the prevalence of BOO was higher in our cohort.

The prevalence varies not only due to different target populations, but also as a result of the different diagnostic criteria for BOO. Although Qmax and Pdet.Qmax are the common criteria, the normal values for Qmax vary between 11 to 15 ml/s^12,13^ and for Pdet.Qmax between 20 to 50 cmH_2_O^13,14^. Soloman et al. proposed the female BOOI (BOOIf = Pdet.Qmax − 2.2*Qmax), which correlated with the probability of BOO^15^. In the current study, we used radiological obstruction as the diagnostic standard^5^. Although the BOOI was higher in the BOO group (−0.41 vs. −4.64) from our results, these pressure-flow-based criteria might not be applicable in patients with DU-like UDS profiles.

The lower Pdet.Qmax and the lower prevalence of DO in the BOO group seem unexpected, as BOO is believed to result in higher detrusor pressure and higher incidence of DO. The notion that “BOO patient should have higher Pdet than non-BOO patients” comes from the classical impression of high-pressure low-flow scenario in common LUTS patients. The concept is based on the assumption that all people can generate high enough detrusor pressure; thus no prior data is available in patients with DU. In follow-up studies of men with DU who underwent TURP, Pdet.Qmax and BCI actually became higher after the relief of obstruction^16,17^. Although this offers only indirect evidence and there is no similar study in women, the statement that “patients with BOO should have higher Pder.Qmax than patients without” might not be applied to all situations. According to the hypothesis of BOO, compensation, and subsequent decompensation of bladder dysfunction^18^, the development of DU usually comes after DO. In the phase of bladder decompensation, DO could become less detectable, resulting in the lower prevalence in the BOO group.

The predominant symptoms and the major etiology of female LUTS are often non-accordant. In women with BOO, the storage symptoms are still very common^9,19^. Reports regarding the symptoms in women with DU are scarce. In a study of UDS of elderly patients, the prevalence of storage and voiding symptoms in women with DU were 55.9% and 23.5%, respectively^20^. Gammie et al. performed a comprehensive comparison of the symptoms and signs in patient with DU and BOO. They found the occurrence of decreased urinary stream, enuresis, and decreased sensation were higher in patients with DU compared to patients with BOO. However, they used a high-pressure low-flow definition for BOO, and the diagnoses of DU and BOO were mutually exclusive under their criteria^21^. In our cohort of DU-like women, symptoms were discernable between BOO and non-BOO patients.

In addition to DU, there are other possible diagnosis from a low-pressure low-flow UDS. For example, some patients might have DO with DU (previously known as DHIC, detrusor hyperreflexia with impaired contractility)^22^. These patients are difficult to treat as the detrusors are overactive in the storage phase but underactive in the voiding phase. As a result, the management for such patients are often symptom directed^23^. Nonetheless, these co-existing conditions do not exclude the possibility of occult BOO. In addition, treating BOO can improve the symptoms and the UDS profiles in women with DU. Medical therapies with alpha-blockers or muscle relaxants are effective in lowering bladder outlet resistance^24,25^. Surgical therapies including transurethral incision of the bladder neck or peri-urethral injection of botulinum toxin are effective in medically refractory patients^26,27^. Therefore, it is important that the presence of BOO is investigated in women with low-pressure low-flow UDS profiles.

The criteria of female BOO by Nitti requires a sustained detrusor contraction. Whether abdominal straining should be taken into consideration when diagnosing BOO remains debatable. Because most patients in this cohort could not achieve sustained detrusor contractions, abdominal straining was often applied to overcome urethral resistance. In such patients, the widening of bladder neck and urethral sphincter indicates non-obstruction, but the absence of bladder neck opening under abdominal straining does not necessarily indicate obstruction. Therefore, the prevalence of BOO under our definition could be over-estimated. The possibility of over-diagnosis can be reflected by the fact that all of the UDS findings in the BOO group (poorer bladder sensation, lower Pdet.Qmax, lower Qmax, smaller VV, larger PVR, and fewer DO) could be well-explained by severe DU alone, in the absence of BOO.

There were three major limitations of our study. First, the inclusion criteria were arbitrary. As there is no global consensus on the criteria of DU in women, we used Pdet.Qmax < 20 cmH2O and Qmax < 15 mL/s as the cut-off values^7^, which should be low enough in most researchers’ viewpoints. We excluded patients with medical histories highly suggestive of neurogenic BOO (spinal cord injury, multiple sclerosis, and myelomeningocele), sphincter deficiency or iatrogenic BOO (stress urinary incontinence), and specific bladder conditions (interstitial cystitis, and ketamine cystitis). Still, the enrolled cohort consisted of relatively heterogeneous groups of patients that have DU and potentially some other concomitant lower urinary tract disorders. Generally, stroke causes DU in the acute phase and then DO in the long-term. Diabetes causes DO initially but leads to DU eventually. Dementia can impair bladder sensation and continence. ESRD causes reduced urine production. Because these diseases have no direct links to BOO and the prevalence were low in both groups, their influences should be small. Second, due to the retrospective nature of the study, the follow-ups of these cases were incomplete. Although most patients received treatments according to their VUDS diagnoses, the responses were not assessable. Third, over-diagnosis of BOO is possible based on our criteria. In patients with co-existing DU and BOO, we cannot be sure which contributes more to their symptoms. However, as treatment is largely limited for DU, the manageable BOO warrants more attention. The profound bladder damage is reversible after the relief of BOO in men, and could probably be so in women.

Despite the limitations, and accepting that our described diagnostic method is valid, our study pointed out several important findings. First, BOO is prevalent in women presumably having DU. Second, obstructive symptoms are reliable indicators in these patients. Finally, simple parameters, such as Qmax and PVR, are useful predictive factors for BOO. In these patients with Pdet.Qmax < 20 cmH2O, BOO is characterized with an even lower Pdet.Qmax. VUDS is indicated in patients with these factors to confirm the diagnosis.

## Conclusions

In women with non-neurogenic LUTS and low-pressure low-flow UDS profiles, there were significant differences in the symptoms and the UDS parameters between the obstructive and the non-obstructive groups. Simple but important indicators suggesting BOO include large PVR, slow Qmax, and predominantly obstructive symptoms.

## Data Availability

All data generated or analyzed during this study are included in this published article.
